# IMPLEMENTING FUNCTION-FOCUSED CARE IN THE HOSPITAL: LESSONS LEARNED

**DOI:** 10.1093/geroni/igac059.1536

**Published:** 2022-12-20

**Authors:** Marie Boltz

**Affiliations:** Penn State, Pennsylvania State University, Pennsylvania, United States

## Abstract

The mentorship provided by the nurse interventionist is an important implementation strategy to support Function-focused care adoption in hospitalized older adults with dementia. The nurse interventionist works with unit champions and interdisciplinary stakeholder teams over a 12 month period. The first interaction includes using a brainstorming approach to develop unit-specific goals to support the integration of function-focused care into routine care delivery. Implementation strategies at the unit level include flexible staff training, involving staff in evaluations of care interactions, targeting pragmatic measures, and a feedback loop. Based on content analysis of field notes we will describe stakeholder goals established, action plans, and barriers and facilitators to meet goals in four hospital units. Key findings include the influence of leadership support, communication strategies, engagement of direct care staff, interdisciplinary collaboration, access to human and material resources, and contextual factors within the hospital setting (including pandemic-related challenges).

